# Supplementary material for: A Novel Cell-Free DNA Fragmentomic Assay and Its Application for Monitoring Disease Progression in Real Time for Stage IV Cancer Patients
Source: Cancers (Basel). 2025 Nov 6;17(21):3583. doi: 10.3390/cancers17213583 (PMC12606779; doi:10.3390/cancers17213583)
Supplement: Supplementary file 1 [file cancers-17-03583-s001.zip › cancers-3946192-supplementary.pdf]

Supplementary Materials for:
A Novel Cell-Free DNA Fragmentomics Assay and Its Application for Monitoring Disease Progression in Real-Time for Stage IV Cancer Patients

Table S1. Participant Summary.

**Participant Outcome**	#	%
Protocol violations, incomplete follow-up	4	2.8%
Withdrawn / Death / Hospice	12	8.3%
Exceeded 72-hour sample stability threshold	2	1.4%
Completed	128	88.9%
Total	146	100.0%
Age	128	100.0%
<60	41	32.0%
≥60	87	68.0%
Average	62.75	
Sex	128	100.0%
Female	73	57.0%
Male	55	43.0%
Race and Ethnicity	128	100.0%
Asian	2	1.6%
Black	21	16.4%
Hispanic	*0*	*0%*
Non-Hispanic	*19*	*14.8%*
Not Reported	*2*	*1.6%*
White	102	79.7%
Hispanic	*1*	*0.8%*
Non-Hispanic	*95*	*74.2%*
Not Reported	*6*	*4.7%*
Other	3	2.3%
Tumor Type	128	100.0%
Breast	20	15.6%
Colorectal	50	39.1%
Lung	58	45.3%
NSCLC	*50*	*39.1%*
SCLC	*8*	*6.3%*
Therapy Type	128	100.0%
Chemotherapy	63	49.2%
Targeted Therapy	7	5.5%
Targeted Therapy + Chemotherapy	7	5.5%
Immunotherapy	14	10.9%
Immunotherapy + Chemotherapy	37	28.9%

Table S2. Analytic Validation summary.

Metric	Value
Limit of Blank (LoB)	95th percentile:<br>• LoB for >80bp cfDNA: 0.06614 pg/μL.<br>• LoB for >265bp cfDNA-0.03602 pg/μL
Limit of Detection (LoD)	LoD=LoB+CβSD:<br>• LoD for >80bp cfDNA: 0.138 pg/μL.<br>• LoD for >265bp cfDNA: 0.139 pg/μL.
Limits of Quantitation (LoQ)	• Upper: LoQ for >80bp and >265bp cfDNA: 20ng/μL.<br>• Lower: LoQ for >80bp and >265bp cfDNA: 0.0006 ng/μL.<br>Upper and lower LoQs are set to the highest and the lowest DNA standard concentration, respectively.
Linearity and Reportable Range	• Upper: LoQ for >80bp and >265bp cfDNA: 20ng/μL.<br>• Lower: LoQ for >80bp and >265bp cfDNA: 0.0006 ng/μL.<br>The upper and lower LoQs are set to the highest and lowest DNA standard concentrations, respectively.

Table S3. qPCR-measured plasma concentrations (copies/mL) of 80 bp (SM1, SM2) and 105 bp (MM1, MM2) amplicons from first and second blood draws, respectively. Derived parameters include short fragment load (Frag1 = SM1 – MM1; Frag2 = SM2 – MM2), changes over time (FragDiff, MMDiff), and Progression Score (PS). Disease progression status is reported by imaging (PD or Non-PD) and molecular criteria (PS-based).

Table S3. Analysis Data (n = 128).

SubjectId	SM1	MM1	SM2	MM2	Frag1	Frag2	FragDiff	MMDiff	PS	Imaging	Molecular
002001	122.1	99.8	39.0	35.0	22.32	4.03	-18.28	-64.84	0.0	Non-PD	Non-PD
002002	16.0	14.5	12.8	11.0	1.51	1.79	0.28	-3.57	7.6	Non-PD	Non-PD
002004	27.8	25.2	30.4	27.2	2.61	3.22	0.61	1.95	7.7	Non-PD	Non-PD
002005	15.8	15.4	19.1	17.7	0.32	1.45	1.13	2.22	13.5	Non-PD	Non-PD
002006	11.9	11.1	17.1	16.8	0.78	0.34	-0.44	5.69	1.7	Non-PD	Non-PD
002009	6.4	6.1	6.9	7.1	0.33	0.00	-0.33	1.04	2.7	Non-PD	Non-PD
002010	21.0	17.9	17.8	14.1	3.12	3.65	0.54	-3.75	10.2	PD	Non-PD
002012	8.4	7.4	9.8	9.1	0.98	0.64	-0.34	1.73	2.6	Non-PD	Non-PD
002013	18.3	17.6	19.0	19.8	0.69	0.00	-0.69	2.16	1.6	Non-PD	Non-PD
002014	9.3	8.1	9.6	9.4	1.19	0.26	-0.93	1.23	1.3	Non-PD	Non-PD
002015	62.4	46.0	64.5	46.8	16.43	17.65	1.22	0.79	16.0	Non-PD	Non-PD
002016	66.4	62.6	65.4	52.7	3.83	12.78	8.95	-9.91	100.0	PD	PD
002017	10.5	9.4	5.6	4.4	1.14	1.23	0.09	-4.95	6.7	Non-PD	Non-PD
002019	447.3	369.6	1217.3	1092.8	77.67	124.48	46.81	723.18	95.4	PD	PD
002020	13.3	11.6	18.5	17.2	1.70	1.29	-0.41	5.63	1.8	Non-PD	Non-PD
002021	194.9	162.1	190.6	143.1	32.79	47.45	14.66	-18.96	100.0	PD	PD
002022	25.4	20.6	258.1	253.1	4.86	4.94	0.08	232.59	0.0	Non-PD	Non-PD
002023	47.6	50.6	19.8	18.6	0.00	1.16	1.16	-32.03	64.6	PD	Non-PD
002024	13.3	11.9	35.4	32.4	1.33	3.03	1.71	20.47	7.9	Non-PD	Non-PD
002026	28.9	23.9	19.3	15.3	4.99	4.00	-0.99	-8.62	2.4	Non-PD	Non-PD
002028	35.6	37.6	36.9	36.3	0.00	0.62	0.62	-1.32	9.7	Non-PD	Non-PD
002029	26.6	25.0	19.6	17.4	1.61	2.17	0.56	-7.59	13.4	Non-PD	Non-PD
002035	21.7	20.1	24.9	24.0	1.61	0.94	-0.67	3.83	1.5	Non-PD	Non-PD

003003	23.5	20.2	112.4	95.0	3.29	17.41	14.11	74.83	100.0	PD	PD
004001	11.6	10.0	9.6	9.6	1.59	0.03	-1.56	-0.39	0.7	Non-PD	Non-PD
004002	45.9	41.5	34.3	32.2	4.38	2.12	-2.26	-9.30	0.5	Non-PD	Non-PD
004003	47.7	39.2	15.4	13.3	8.47	2.08	-6.39	-25.92	0.0	Non-PD	Non-PD
004004	10.8	9.3	9.6	9.4	1.48	0.18	-1.30	0.15	0.9	Non-PD	Non-PD
004005	12.1	10.4	7.8	7.2	1.70	0.61	-1.09	-3.22	1.5	Non-PD	Non-PD
004006	120.4	120.4	78.3	79.3	0.00	0.00	0.00	-41.18	45.7	Non-PD	Non-PD
004010	15.5	13.0	37.0	35.3	2.50	1.71	-0.79	22.28	0.4	Non-PD	Non-PD
004011	9.5	9.0	10.2	10.6	0.50	0.00	-0.50	1.65	2.1	Non-PD	Non-PD
004012	11.4	10.5	13.8	13.1	0.95	0.67	-0.29	2.64	2.6	Non-PD	Non-PD
004013	139.0	118.6	47.9	43.2	20.37	4.75	-15.61	-75.47	0.0	Non-PD	Non-PD
004014	11.8	11.4	14.8	12.7	0.40	2.10	1.70	1.29	24.9	Non-PD	Non-PD
004016	11.2	8.0	8.4	6.9	3.19	1.49	-1.70	-1.15	0.6	Non-PD	Non-PD
004017	8.4	7.9	9.7	9.2	0.41	0.45	0.04	1.28	4.1	Non-PD	Non-PD
004019	20.8	17.9	183.6	181.7	2.93	1.99	-0.94	163.77	0.0	Non-PD	Non-PD
004020	35.7	31.0	36.1	31.0	4.72	5.13	0.41	0.03	7.0	Non-PD	Non-PD
004021	40.5	37.5	17.1	15.6	2.94	1.52	-1.41	-21.92	3.6	Non-PD	Non-PD
004022	16.5	13.7	16.7	14.6	2.74	2.05	-0.69	0.88	1.8	Non-PD	Non-PD
004023	31.8	27.9	15.5	15.4	3.91	0.09	-3.82	-12.51	0.1	Non-PD	Non-PD
004024	22.1	18.3	67.6	67.6	3.78	0.02	-3.77	49.29	0.0	Non-PD	Non-PD
004025	10.8	10.5	10.4	10.5	0.30	0.00	-0.30	-0.07	3.1	Non-PD	Non-PD
004026	14.8	15.1	193.5	192.6	0.00	0.90	0.90	177.50	0.0	Non-PD	Non-PD
004029	16.9	14.7	13.7	12.0	2.28	1.75	-0.53	-2.65	2.8	Non-PD	Non-PD
004031	6.8	6.0	7.1	6.4	0.83	0.72	-0.10	0.35	3.7	Non-PD	Non-PD
005001	84.1	69.1	64.1	62.3	15.00	1.80	-13.20	-6.80	0.0	Non-PD	Non-PD
005003	6.9	6.8	6.2	5.7	0.10	0.44	0.34	-1.07	6.9	Non-PD	Non-PD
005004	28.8	24.9	58.0	55.6	3.99	2.34	-1.65	30.78	0.1	Non-PD	Non-PD
005006	15.0	11.8	42.1	37.3	3.24	4.76	1.52	25.55	4.6	Non-PD	Non-PD
005008	16.0	12.3	5.7	4.5	3.66	1.22	-2.44	-7.81	0.4	Non-PD	Non-PD
005009	25.8	23.5	33.9	30.9	2.33	3.07	0.73	7.41	6.2	Non-PD	Non-PD
005010	20.6	17.7	8.9	8.2	2.91	0.77	-2.14	-9.53	0.6	Non-PD	Non-PD
005012	17.3	16.9	34.8	32.3	0.41	2.50	2.08	15.37	16.4	Non-PD	Non-PD
005013	9.9	10.9	19.3	17.0	0.00	2.30	2.30	6.12	33.1	Non-PD	Non-PD
005015	39.8	30.7	33.7	30.4	9.07	3.32	-5.75	-0.28	0.0	Non-PD	Non-PD
005016	34.7	30.0	30.1	28.8	4.71	1.33	-3.38	-1.21	0.1	Non-PD	Non-PD
005017	8.8	8.2	15.4	13.7	0.64	1.62	0.98	5.55	9.2	Non-PD	Non-PD
005018	21.1	20.5	15.5	13.8	0.66	1.78	1.12	-6.70	22.4	Non-PD	Non-PD
005019	520.3	456.3	84.1	77.2	63.99	6.88	-57.11	-379.09	0.0	Non-PD	Non-PD
005020	22.5	20.5	18.9	17.0	1.96	1.86	-0.10	-3.53	4.9	Non-PD	Non-PD
005022	19.6	18.7	17.5	16.0	0.91	1.52	0.61	-2.76	10.4	Non-PD	Non-PD
005023	14.3	12.0	13.7	11.5	2.33	2.25	-0.08	-0.55	4.1	PD	Non-PD
005024	272.4	250.8	121.3	104.5	21.67	16.73	-4.94	-146.26	77.3	PD	Non-PD
005025	18.3	17.7	28.6	26.8	0.66	1.87	1.21	9.09	9.5	Non-PD	Non-PD
005027	10.6	9.7	12.0	10.8	0.92	1.20	0.28	1.08	5.6	PD	Non-PD
006001	120.9	107.1	27.3	23.9	13.78	3.43	-10.35	-83.26	0.0	Non-PD	Non-PD
008001	8.5	8.3	7.5	7.3	0.16	0.18	0.01	-1.01	4.7	Non-PD	Non-PD
008002	16.0	13.9	21.2	20.6	2.16	0.67	-1.49	6.71	0.4	Non-PD	Non-PD
008003	12.7	10.0	48.1	46.6	2.74	1.50	-1.24	36.57	0.1	Non-PD	Non-PD
008004	130.6	125.6	35.8	31.6	4.99	4.22	-0.77	-94.03	93.3	PD	PD
009001	11.6	10.8	19.4	18.2	0.80	1.23	0.43	7.40	4.3	Non-PD	Non-PD

010001	32.7	25.1	33.7	31.5	7.59	2.19	-5.39	6.38	0.0	Non-PD	Non-PD
010002	131.7	133.0	22.4	18.0	0.00	4.40	4.40	-114.96	100.0	PD	PD
010003	4.6	4.4	60.0	59.6	0.26	0.41	0.15	55.26	0.1	Non-PD	Non-PD
010004	21.1	18.6	18.5	18.7	2.51	0.00	-2.51	0.03	0.2	Non-PD	Non-PD
010005	44.9	42.6	31.4	31.0	2.28	0.39	-1.88	-11.56	1.0	Non-PD	Non-PD
010006	33.7	30.0	9.0	8.9	3.68	0.07	-3.61	-21.11	0.2	Non-PD	Non-PD
010007	12.2	10.6	8.8	6.7	1.64	2.10	0.46	-3.89	9.5	PD	Non-PD
010008	3.6	3.3	3.8	3.6	0.28	0.28	0.00	0.27	4.2	Non-PD	Non-PD
010009	143.0	139.8	16.0	15.4	3.26	0.56	-2.71	-124.34	91.7	PD	PD
010010	141.9	118.6	149.7	115.7	23.34	34.03	10.68	-2.91	100.0	PD	PD
010011	84.8	79.7	33.1	26.3	5.02	6.79	1.76	-53.44	94.6	PD	PD
010012	17.3	16.9	20.7	19.9	0.40	0.80	0.40	2.97	5.6	Non-PD	Non-PD
010014	34.8	30.1	34.8	29.9	4.74	4.88	0.14	-0.17	5.1	Non-PD	Non-PD
010015	21.3	19.6	16.6	12.9	1.70	3.70	2.00	-6.71	46.0	PD	Non-PD
010016	12.6	11.2	11.5	10.8	1.45	0.63	-0.81	-0.32	1.7	Non-PD	Non-PD
010017	142.6	136.6	55.8	48.5	6.01	7.31	1.30	-88.06	99.1	PD	PD
010018	18.3	17.4	18.8	19.5	0.87	0.00	-0.87	2.02	1.3	Non-PD	Non-PD
010019	45.7	42.5	75.7	75.9	3.20	0.00	-3.20	33.50	0.0	Non-PD	Non-PD
010020	21.1	19.6	23.8	19.5	1.49	4.30	2.81	-0.14	59.0	Non-PD	Non-PD
010021	28.5	26.1	21.4	20.6	2.46	0.84	-1.62	-5.46	0.9	Non-PD	Non-PD
010022	11.6	11.3	12.4	12.7	0.29	0.00	-0.29	1.44	2.8	Non-PD	Non-PD
010023	27.6	24.5	20.8	17.0	3.11	3.84	0.73	-7.53	15.9	Non-PD	Non-PD
010024	12.3	11.6	18.6	16.0	0.75	2.59	1.84	4.44	24.1	Non-PD	Non-PD
010025	10.9	10.6	14.7	12.7	0.34	2.02	1.68	2.11	23.4	Non-PD	Non-PD
010027	18.3	16.4	20.8	17.6	1.86	3.22	1.36	1.14	18.1	Non-PD	Non-PD
010028	24.0	24.0	29.2	25.7	0.03	3.47	3.44	1.70	73.3	PD	Non-PD
010029	36.0	33.9	41.8	33.8	2.11	8.08	5.97	-0.12	98.6	PD	PD
010030	72.9	59.3	80.0	64.2	13.67	15.77	2.11	4.94	29.8	Non-PD	Non-PD
010033	10.2	8.6	4.3	3.8	1.52	0.51	-1.02	-4.80	1.8	Non-PD	Non-PD
010035	12.8	11.1	18.1	18.2	1.68	0.00	-1.68	7.17	0.3	Non-PD	Non-PD
010040	9.6	9.1	10.3	8.4	0.53	1.89	1.35	-0.69	20.0	Non-PD	Non-PD
010041	25.2	21.8	41.3	33.7	3.39	7.58	4.19	11.88	77.1	PD	Non-PD
010042	13.7	11.2	19.7	17.8	2.50	1.90	-0.61	6.61	1.3	Non-PD	Non-PD
010043	42.7	42.4	15.4	15.9	0.30	0.00	-0.30	-26.43	16.9	Non-PD	Non-PD
010047	20.1	18.3	23.0	21.3	1.80	1.73	-0.08	2.95	3.2	Non-PD	Non-PD
010048	36.8	35.2	15.1	13.5	1.57	1.66	0.09	-21.71	19.0	Non-PD	Non-PD
010052	22.0	20.3	17.7	18.2	1.63	0.00	-1.63	-2.17	0.7	Non-PD	Non-PD
010053	22.8	19.1	26.1	23.7	3.66	2.45	-1.21	4.57	0.7	Non-PD	Non-PD
010055	11.4	10.0	14.6	12.9	1.48	1.64	0.17	2.96	4.3	PD	Non-PD
010056	23.6	20.1	30.7	25.5	3.51	5.17	1.67	5.41	19.3	Non-PD	Non-PD
010057	52.3	42.5	33.0	24.4	9.81	8.62	-1.19	-18.07	3.7	Non-PD	Non-PD
010058	27.5	24.3	11.8	10.0	3.15	1.81	-1.35	-14.29	2.3	Non-PD	Non-PD
010059	13.6	10.9	12.4	10.2	2.73	2.19	-0.54	-0.68	2.4	Non-PD	Non-PD
010060	153.1	121.2	134.7	110.7	31.93	24.05	-7.87	-10.52	0.0	Non-PD	Non-PD
011002	21.9	19.3	10.7	9.2	2.54	1.53	-1.01	-10.12	2.6	Non-PD	Non-PD
011003	16.3	13.0	16.7	15.0	3.29	1.71	-1.59	2.00	0.6	Non-PD	Non-PD
011004	41.2	33.9	39.5	31.5	7.32	7.99	0.67	-2.42	10.9	Non-PD	Non-PD
011005	9.0	7.5	6.8	5.7	1.51	1.13	-0.38	-1.77	3.1	Non-PD	Non-PD
011006	18.7	15.5	34.8	31.6	3.18	3.17	-0.01	16.13	1.4	Non-PD	Non-PD
011008	12.4	11.9	18.1	17.3	0.52	0.77	0.25	5.38	4.0	Non-PD	Non-PD

011010	5.9	5.5	10.4	9.8	0.48	0.57	0.09	4.36	3.6	Non-PD	Non-PD
011012	13.7	12.3	47.5	44.6	1.40	2.90	1.50	32.28	2.8	Non-PD	Non-PD
011015	6013.8	5341.5	85.9	80.8	672.29	5.05	-667.25	-5260.64	0.0	Non-PD	Non-PD
011016	166.1	140.8	100.4	85.7	25.36	14.71	-10.65	-55.10	0.0	Non-PD	Non-PD
017001	38.9	33.1	20.5	17.0	5.76	3.50	-2.27	-16.12	0.9	Non-PD	Non-PD

Table S4 presents the impact of non-cancer clinical conditions on the Progression Score (PS) assay. "PS Change" is defined as the difference between the PS score (0–100 scale) of a patient with an acute clinical condition and the mean PS score of nine healthy volunteers (baseline). A PS change may be positive or negative depending on the timing of the condition relative to blood sampling. A positive PS Change indicates a higher PS score relative to healthy controls, potentially mimicking progression-like signals, while a negative or zero PS Change suggests minimal impact.

Table S4. Impact of Non-Cancer Clinical Conditions on Progression Score (PS).

Events 12. days of blood draw).	n	PS Change
Acute Stroke	3	100.0
Asthma	2	4.5
COPD exacerbation	2	43.4
COPD - hydroxyurea	1	98.1
Diabetes out of control, diabetic ketoacidosis	3	26.0
Inflammatory Bowel Disease	4	47.1
Myocardial Infarction	2	100.0
Rheumatoid Arthritis	3	14.4
Rheumatoid Arthritis - methotrexate	2	87.2
Seizure	3	45.0
Viral Infection	5	15.1
